# Characterization of vascular strain during *in-vitro *angioplasty with high-resolution ultrasound speckle tracking

**DOI:** 10.1186/1742-4682-7-36

**Published:** 2010-08-20

**Authors:** Prashant Patel, Rohan Biswas, Daewoo Park, Thomas J Cichonski, Michael S Richards, Jonathan M Rubin, Sem Phan, James Hamilton, William F Weitzel

**Affiliations:** 1 Department of Internal Medicine, University of Michigan, Ann Arbor, MI, USA; 2 Department of Biomedical Engineering, University of Michigan, Ann Arbor, MI, USA; 3 Department of Electrical and Computer Engineering, University of Rochester, Rochester, NY, USA; 4 Department of Radiology, University of Michigan, Ann Arbor, MI, USA; 5 Department of Pathology, University of Michigan, Ann Arbor, MI, USA; 6 Epsilon Imaging Inc., Ann Arbor, MI, USA

## Abstract

**Background:**

Ultrasound elasticity imaging provides biomechanical and elastic properties of vascular tissue, with the potential to distinguish between tissue motion and tissue strain. To validate the ability of ultrasound elasticity imaging to predict structurally defined physical changes in tissue, strain measurement patterns during angioplasty in four bovine carotid artery pathology samples were compared to the measured physical characteristics of the tissue specimens.

**Methods:**

Using computational image-processing techniques, the circumferences of each bovine artery specimen were obtained from ultrasound and pathologic data.

**Results:**

Ultrasound-strain-based and pathology-based arterial circumference measurements were correlated with an R^2 ^value of 0.94 (p = 0.03). The experimental elasticity imaging results confirmed the onset of deformation of an angioplasty procedure by indicating a consistent inflection point where vessel fibers were fully unfolded and vessel wall strain initiated.

**Conclusion:**

These results validate the ability of ultrasound elasticity imaging to measure localized mechanical changes in vascular tissue.

## Introduction

Peripheral vascular disease is a widespread problem in the United States [1-3]. Current treatment options aimed at tissue revascularization are effective; however, practitioners continue to face the underlying disease process of neointimal hyperplasia leading to restenosis [4-7]. Ultrasonography has been used for graft surveillance to detect stenotic lesions [8]. The use of local elasticity imaging has provided more accurate estimates of the biomechanical properties of tissue by directly measuring intramural strain. Ultrasonography with phase-sensitive speckle-tracking algorithms is increasingly used as a robust, noninvasive tool for assessing the mechanical and elastic properties of subsurface structures, including vascular tissue [9-11]. Recent investigation indicates the potential of using Doppler strain rate imaging to clinically assess elastic properties of the vessel wall in patients with coronary artery disease [12]. Beyond the direct strain measurements that have been employed to date, ultrasound elasticity imaging has the potential to distinguish simple tissue motion or "translation" from the strain or "deformation" that we investigate in this study.

Since angioplasty is a common treatment for stenosis and results in changes in the arterial fiber anatomy of the tunica media as the angioplasty balloon expands, we investigated the ultrasound elasticity imaging characteristics of angioplasty in the laboratory setting. We hypothesized that elasticity imaging may detect different strain patterns as the arterial fibers unfold during balloon expansion. We further hypothesized that normal strain and shear strain may indicate physical changes in fiber architecture corresponding to the angioplasty process. To evaluate the ability of ultrasound elasticity imaging to detect definable histologic changes induced during angioplasty, we compared ultrasound strain measurements of bovine artery specimens with the physical characteristics of the vessel obtained on pathology tissue specimen examination.

## Methods

### Elasticity Imaging Data

**Figure 1:**
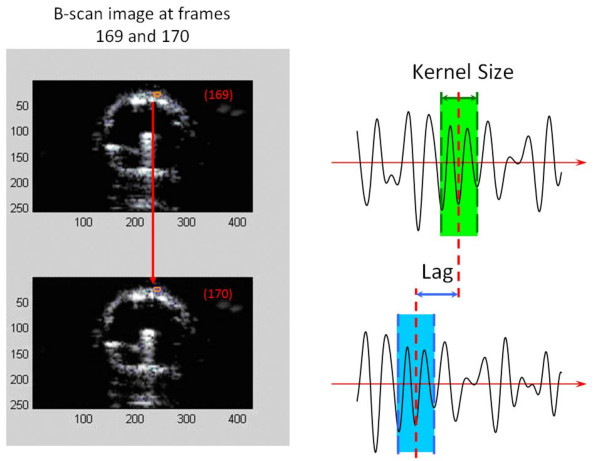
**The displacement of the vessel wall from frame to frame observed using the "lag" distance in the underlying ultrasound signal**. These displacements are estimated using correlation-based algorithms and phase-sensitive speckle tracking.

High-resolution imaging data can be obtained using radio frequency (RF) ultrasound signals containing speckle information to accurately track the motion of structures within an imaged object such as the lumen wall of an artery [13,14]. The first step in this process is to estimate the motion, or displacement, of the object from frame to frame. The frames need not be adjacent. The displacement of the object in the ultrasound images is estimated using a two-dimensional, correlation-based, phase-sensitive speckle-tracking technique [15]. Figure 1 illustrates the displacement "lag" from one frame to the next calculated using the underlying RF ultrasound signal. The axial displacement is then further refined by determining the zero-crossing position of the phase of the analytic signal correlation. Strain values are determined by numerically calculating the spatial derivatives (gradients) of the displacement values.

The components of strain were determined according to the location of the arterial wall. The two principal strain components were axial strain, which is the strain along the beam direction, and lateral strain, which is perpendicular to the axial strain. The derivative, with respect to time, of the displacement provides the strain. For two-dimensional speckle tracking, this process is repeated multiple times for each beam and between adjacent beams that comprise the image. For our study, the axial and lateral displacements were calculated at the position of the maximum correlation coefficient, using a correlation kernel size approximately equal to the speckle spot. The axial displacement estimate was then further refined by determining the phase-zero crossing position of the analytic signal correlation. A spatial filter twice as large as the kernel size was used to enhance signal-to-noise ratio for better spatial resolution. A weighted correlation window was used with spatial filtering of adjacent correlation functions to reduce frame-to-frame displacement error. To support the calculation of strain, inter-frame motion of reference frame pixels was integrated to produce the accumulated tissue displacement. Spatial derivatives of the displacements were calculated in a region of the artery to estimate the radial normal strain. All strain values were measured in the axial direction, where resolution is at least an order of magnitude greater than that in the lateral direction. Thus, the axial strain is more accurate due to the direction of the beam.

Ultrasound data and video B-scans were obtained for four of five bovine carotid artery specimens and used to determine the vessel diameter and path-length data (Artegraft^®^, North Brunswick, NJ, USA). The fifth artery specimen served as the control, which would indicate the "plasty," or change in fiber architecture, of the other samples. These reasonably uniform tissue samples were preserved in 1% propylene oxide and 40% aqueous U.S.P. ethyl alcohol. Because this *in vitro *model is highly idealized, it is limited in accounting for the behavior of diseased vessels which may be hyperplastic or atherosclerotic. However, the samples are produced for clinical use in vascular bypass and dialysis access construction, making them an excellent vascular substrate for our angioplasty study.

A WorkHorse™ II (AngioDynamics, Queensbury, NY, USA) angioplasty balloon (10-mm diameter by 4-cm length) was inserted into each artery. The standard, non-compliant balloon was expanded manually using linearly increasing pressure while observing the pressure sensor reading during ultrasound data capture. Specimens were suspended in an ultrasound water tank containing physiologic (9%) saline solution. Imaging was performed using a Siemens Sonoline Elegra scanner (SSN4363, Deerfield, IL, USA) with a 7.5-MHz linear ultrasound transducer fixed in a harness for data collection while the angioplasty balloon was inflated from 0 to 5 atm of pressure in all experimental specimens. The uninflated pressures were transmitted to the wall during inflation by balloon unfolding. However, the interaction between the unfolding balloon and the arterial wall is likely to be complicated, and some of the friction between the balloon surface and the intima is zero. Because we were unable to measure these effects, they were not included in the experimental method. Once the balloon was inflated, no further pressure in the balloon was transmitted to the arterial wall. Uneven stress due to balloon folding was a limitation of our experimental method; however, this is the way angioplasty is conducted in the clinical setting. The balloon-inflated vessels were fully expanded at 2 atm. Real-time RF data were collected and processed off-line using computational techniques for each artery.

Four regions of interest (ROIs) were selected on the leading edge of the top wall of each vessel. They were sequentially ordered based on their position relative to the center of the leading edge. These ROIs were tracked for observing strain patterns during angioplasty balloon deformation in the ultrasound B-scan image and determined the regions on the vessel wall where longitudinal strain, shear strain and average data quality index (DQI) would be calculated on the basis of the radial displacement of the lumen wall. The longitudinal strain was calculated as the gradient of the longitudinal displacement (derivative of the displacement) along the ultrasound beam, and the shear strain was calculated as the partial derivative of the longitudinal displacement (movement along the ultrasound beam) across the beams. The DQI is the measure of the frame-to-frame correlation, using the phase-sensitive cross-correlation methods previously developed [15]. The DQI is therefore a measure of the accuracy of motion tracking between frames, used to quantify the quality of the data. A maximal value of 1 indicates the highest level of tracking reliability. Young's moduli were obtained for the ROIs and compared against reported normal physiologic moduli calculated for similar vascular tissue.

The two-dimensional longitudinal strain is defined as $\varepsilon_{x}=\frac{\partial u_{x}}{\partial x}$, $\varepsilon_{y}=\frac{\partial u_{y}}{\partial y}$ and the two-dimensional shear strain is defined as $\varepsilon_{xy}=\varepsilon_{yx}=\frac{1}{2}\left(\frac{\partial u_{y}}{\partial x}+\frac{\partial u_{x}}{\partial y}\right)$. The $\frac{\partial u_{y}}{\partial x}$ is the normal strain in axial direction, along the beam, and the $\frac{\partial u_{x}}{\partial y}$ is the normal strain in lateral direction. As mentioned before, the axial direction is more accurate than the lateral direction, so shear strain was regarded as $\varepsilon_{xy}=\varepsilon_{yx}=\frac{\partial u_{y}}{\partial x}$.

The Young's modulus of elasticity for the tissue is $E=\frac{\sigma }{\varepsilon }=\frac{FL_{0}}{A_{0}\Delta L}$, ..., where σ is the stress, ε is the strain, F is the applied force in Newtons, L_0 _represents the initial non-deformed length, A_0 _is the cross-sectional area, and ΔL is the change in length. Because the tissue exhibits a non-linear elastic response, the Young's modulus varies depending on the values of L_0 _and ΔL, with the tangent to the stress-strain curve indicating the Young's modulus for a specific L_0_. However, as ΔL approaches zero, inaccuracies in measurement become more pronounced. For our analysis we assumed a linear elastic response (Hooke's Law) over the region of interest, as ΔL is small for angioplasty-induced pressure variations considered in our investigation.

**Figure 2:**
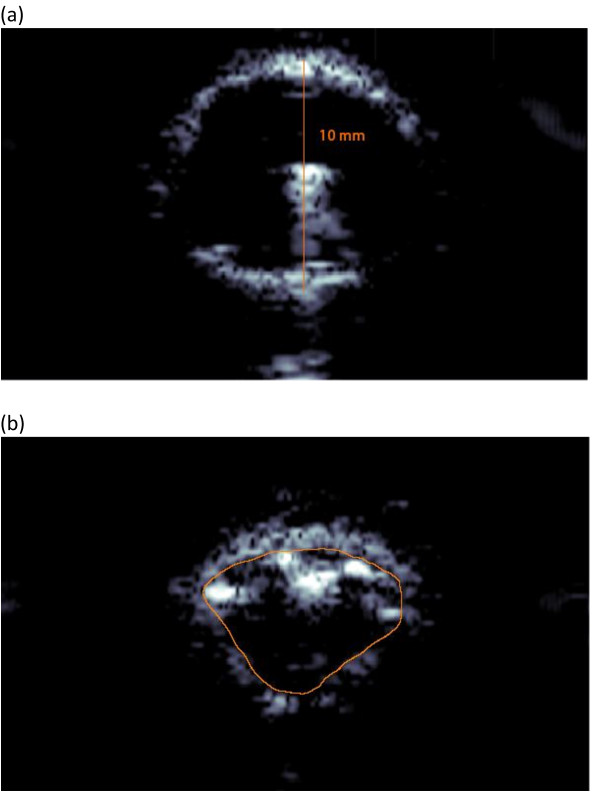
**B-scan images of artery 1**. Given that the diameter of the full-blown angioplasty balloon is 10 mm in the final frame (a), when the artery was stretched to comply with the balloon, the circumference of the vessel wall in the n^th ^frame (b) could be estimated by tracing the inner arterial wall. Note that the superficial spots are parts of the folded balloon.

The ultrasound path lengths were determined using Adobe Illustrator CS2 (AI CS2; Adobe, San Jose, CA, USA) software and the Pathlength plug-in for the program (Telegraphics, Australia) to find the length of each traced fiber given only in the superficial unit of points. Using AI CS2, the *n*^th ^frame and the final frame from the B-mode video were compared for each artery, as seen in Figure 2 for artery 1. The final frame shows the fully inflated angioplasty balloon. Because the diameter of the balloon had a known value of 10 mm, it was possible to use the final frame to obtain the millimeter/points ratio that would be used in calculating elasticity-imaging circumference in millimeters from the ultrasound B-scan image. The circumference of the vessel wall in the *n*^th ^frame, C_n_, was traced and measured using the Pathlength filter and converted into millimeters using the final frame's millimeter/points ratio.

### Pathology Data

**Figure 3:**
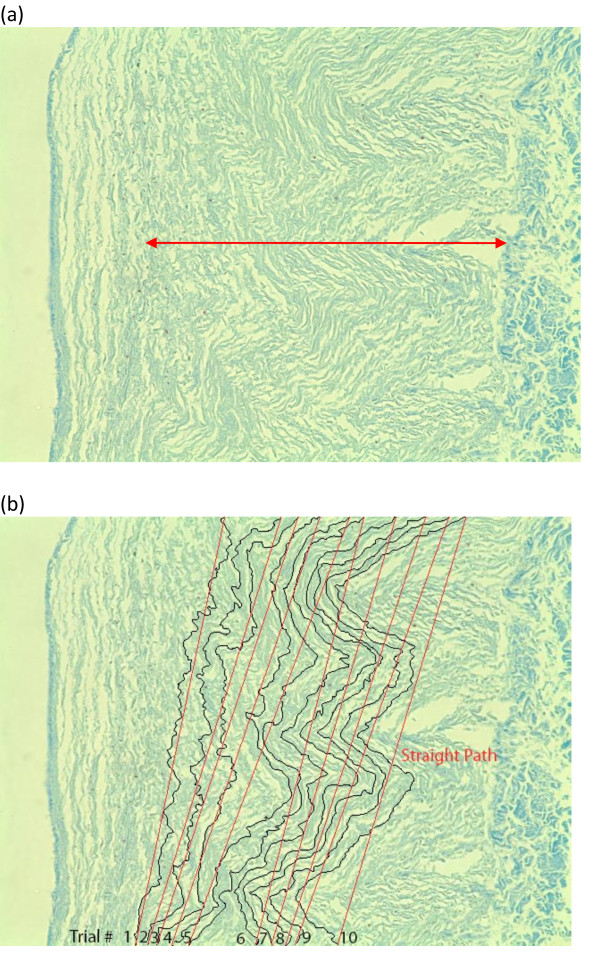
**Magnified histologic image of artery 1**. The region of interest is the tunica media (a). Fibers within this portion of the artery are naturally folded, each following a variable path. The paths of ten fibers were traced in black (b) and lines connecting the origin and endpoint of each path were drawn. The ratio of path length to line length was used to calculate arterial path length.

Five histologic slides were prepared by staining a cross-section of each of the five bovine carotid artery specimens with Masson's trichrome solution (for collagen) to observe the extra-cellular matrix composition. Four magnified images of each specimen were obtained. Figure 3(a) delineates the major region of interest, the tunica media, in these slides.

Using AI CS2 and the Pathlength plug-in, ten separate fibers were traced by hand in each magnified image. Figure 3(b) shows the traced, or "true" paths (black lines) and "straight" paths (red lines), or lines connecting the origin and endpoint of each true path, for several fibers in experimental artery 1. These straight paths were required because the unmagnified photographs would not account for the folded resting state of the artery's media. The ratio of true to straight path length was obtained for each traced fiber.

**Figure 4:**
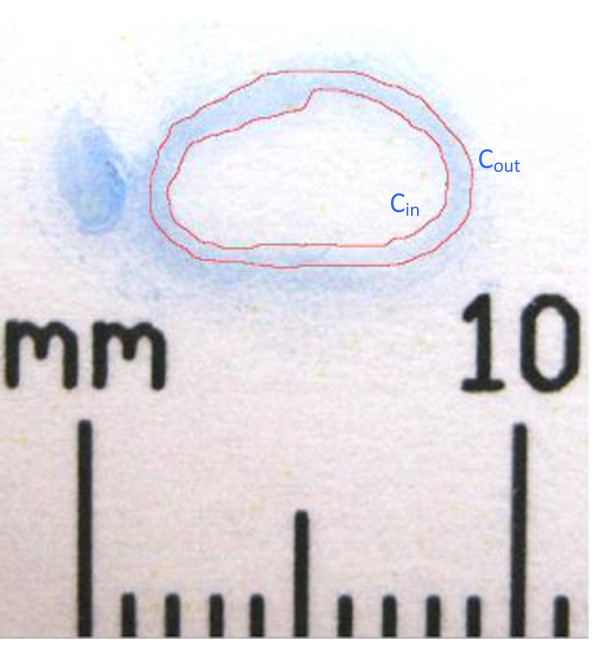
**Digital photograph of cross-section of artery 1**. The circumferences of the inner (C_in_) and outer (C_out_) walls of the artery were obtained.

Figure 4 shows the unmagnified photograph of the slide aligned with a metric ruler as a real-value reference in order to obtain quantitative measurements of the circumference in millimeters. AI CS2 was used to trace and measure the artery's inner (luminal) and outer cross-sectional circumferences (C_in _and C_out_, respectively). Since the true and straight paths from the magnified images were measured close to the center of the media, finding the average of the C_in _and C_out _on the photographs would predict a circumference, C_media_, which was closest to the center of the media. To find the true path length of the entire cross-sectional artery, C_s_, the C_media _was multiplied by each true-to-straight path length ratio from the magnified images with the C_media _circumference value. The mean and standard deviation of the 40 measurements (10 fibers per image × 4 magnified images per specimen) were calculated for each specimen.

## Results

**Figure 5:**
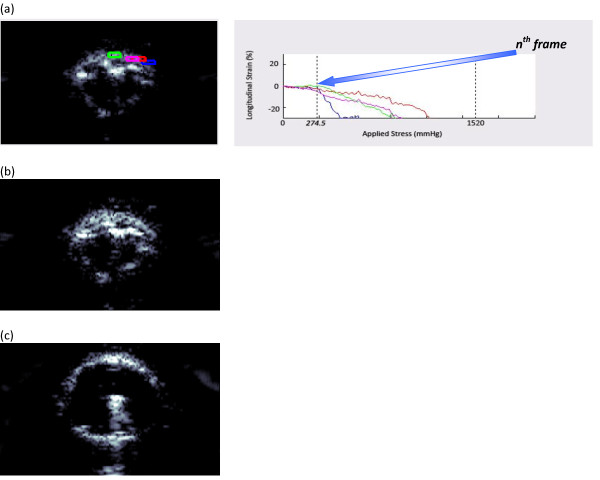
**Key frames in the B-scan images of artery 1**. (a) Four regions of interest in the arterial wall are shown as colored boxes in the B-scan image. The graph of longitudinal strain vs. applied stress for these regions of interest shows a uniform inflection point at the n^th ^frame. The inflection point, or n^th ^frame, was different for each specimen. The initial (b), n^th ^(a), and final (c) frames in the B-scan video confirm changes in vessel circumference during the angioplasty procedure.

Figure 5 shows the ultrasound B-scan images obtained during angioplasty for artery 1. Therefore, the inflection point on the longitudinal strain versus pressure graph indicates the point at which the fibers of the artery had fully unfolded by expansion of the angioplasty balloon. This inflection point arises because the patterns of strain, highlighted by slope, differ between the onset of angioplasty and fiber unfolding, and when unfolded fibers begin experiencing deformation. In general, tissue motion consists of translation and deformation. Elasticity imaging with speckle tracking distinguishes these by measuring the amount of strain occurring during translational motion. The ultrasound B-scan frame where tissue deformation from the angioplasty balloon began is recorded as the inflection point and is shown in Figure 5(a). The inflection point among all four ROIs indicates a homogenous mechanical tissue response along the wall, and was confirmed by cross-analysis with the shear strain versus time graph. Young's moduli data are summarized in Table 1. All of these values were within the normal physiologic Young's modulus range of 200 - 900 kPa [16-18], further confirming elasticity imaging's unique ability to capture localized strain patterns.

**Table 1:** Young's moduli obtained from elasticity imaging for regions of interest (ROIs) in each artery sample

	Artery 1	Artery 2	Artery 3	Artery 4
ROI 1	236.2 kPa	253.2 kPa	224.3 kPa	300.7 kPa
ROI 2	330.1 kPa	227.5 kPa	303.8 kPa	249.5 kPa
ROI 3	495.7 kPa	335.0 kPa	401.6 kPa	241.3 kPa
ROI 4	555.4 kPa	304.6 kPa	512.0 kPa	587.5 kPa

**Figure 6:**
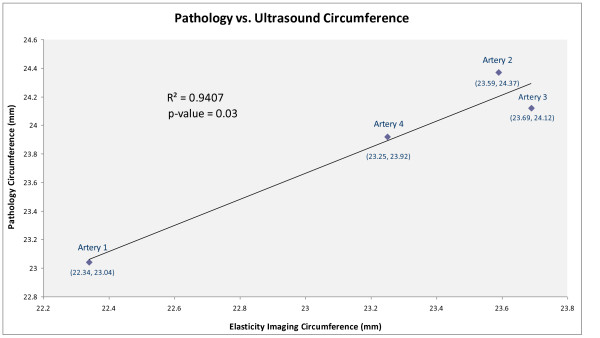
**Comparison of circumference results obtained from pathology and ultrasound measurements**. As seen by the R^2 ^value, the collected data were highly correlated, indicating the accuracy of using elasticity imaging in confirming pathologic data.

The longitudinal strain values for artery 1 had an inflection point at 274.5 mmHg, as shown in Figure 5(a). For comparison, the initial and final B-scan frames are shown in Figures 5(b) and 5(c), respectively. The elasticity-imaging circumference (C_n_) values are compared to the pathology data (C_s_) values in Figure 6. As the figure's trend line indicates, the two sets of circumference data were comparable among the four test specimens, confirming a high degree of accuracy resulting from ultrasound elasticity imaging. Although the sample size was not large and we did not have a group of controls to which we could compare our results, statistical analysis found that the data were highly correlated, with an R^2 ^value of 0.94 and a p-value of 0.03. There was a consistent over or under estimate of ~3%, but more interestingly, there was a high degree of correspondence suggesting a relationship between the pathology and ultrasound elasticity imaging.

These results are quasi-static (at different balloon expansion rates, the circumference value obtained will remain the same) but not reversible, as indicated by the control specimen. The experimental vessel segments were larger in circumference than the control that did not undergo angioplasty. This indicates that some of the fibers experienced "plasty" during balloon inflation and not simply reversible deformation.

## Discussion

In an *in vitro *model of angioplasty, the vessel wall fibers exhibit folding prior to balloon inflation. As the balloon inflates, the vessel expands and undergoes tissue deformation or strain that we were able to observe with elasticity imaging. We further observed changes in the normal strain and shear strain patterns that indicated changes in fiber architecture corresponding to the angioplasty process. These results confirmed elasticity imaging's ability to detect histologically definable characteristics within the vessel. These findings distinguished vascular collagen fiber wall unfolding from fiber deformation or strain during measurements in this *in vitro *vascular model.

Because the synthesis of collagen is accompanied by collagen and elastin cross-linking to provide structural support during vascular healing, and as collagen begins to accumulate, elastin degradation in the media becomes a consistent feature. Consequently, one expects to see increased vessel stiffness as a result of neointimal hyperplasia [19,20].

If wall strain is accurately measured with high resolution, then multiple clinically important *in vivo *characteristics may be determined. First, it may be possible to distinguish radial strain from shear strain, which may differentiate elastic lesions from lesions that actually undergo "plasty," or change in their architecture during balloon inflation, indicating the desired therapeutic effect of the procedure has been achieved. Second, the degree of wall strain coupled with pressure information will allow Young's modulus determination, which may provide quantitative information about the severity of the underlying disease process. Third, local high-resolution strain measurements may provide information about a vessel's risk of rupture and prevent extravasations and other complications. Fourth, the stress-strain relationship during stent placement will provide important information that may help improve the design of stents, and may provide an indicator of risk factors for in-stent re-stenosis.

In this study, a detectable change in the slope of the strain in each artery specimen undergoing angioplasty was clearly observed. This inflection point in strain consistently validated the vessel's structural characteristics after the fibers of the artery had unfolded due to expansion of the angioplasty balloon. Although further study is needed, these results suggest this procedure can detect highly localized mechanical changes in the vessel wall during angioplasty. Future *in vitro *and *in vivo *studies are planned to investigate the ability of ultrasound elasticity imaging to measure the complexities and mechanical properties of the vascular wall.

## Competing interests

The authors declare that they have no competing interests.

## Authors' contributions

All authors contributed to the writing of the manuscript and read and approved the final manuscript. PP, RB, and DWP designed and conducted experimental work, and performed data analysis. TJC participated in data analysis and provided major editorial suggestions. MSR, JMR, and SP performed theoretical background work and experimental design. JH helped design the strain imaging software used in experimental design. WFW conceived and coordinated the study, performed theoretical background work, and participated in experimental work.
